# Inguinal Hernia and Airport Scanners: An Emerging Indication for Repair?

**DOI:** 10.1155/2013/952835

**Published:** 2013-12-03

**Authors:** Vijay Naraynsingh, Shamir O. Cawich, Ravi Maharaj, Dilip Dan

**Affiliations:** Department of Clinical Surgical Sciences, Faculty of Medical Sciences, University of the West Indies, St. Augustine Campus, Trinidad and Tobago

## Abstract

The use of advanced imaging technology at international airports is increasing in popularity as a corollary to heightened security concerns across the globe. Operators of airport scanners should be educated about common medical disorders such as inguinal herniae in order to avoid unnecessary harassment of travelers since they will encounter these with increasing frequency.

## 1. Introduction

Inguinal herniae are common clinical findings in modern surgical practice. Many patients choose to undergo inguinal herniorrhaphy when the minor risks associated with repair are weighed against the potential for the hernia to become complicated. More recently, conservative management has become an accepted therapeutic option for patients with asymptomatic inguinal herniae that are unlikely to strangulate [1, 2]. We report our experience managing a patient with an asymptomatic inguinal hernia who opted for herniorrhaphy with an unusual indication.

## 2. Case Presentation

**Figure 1:**
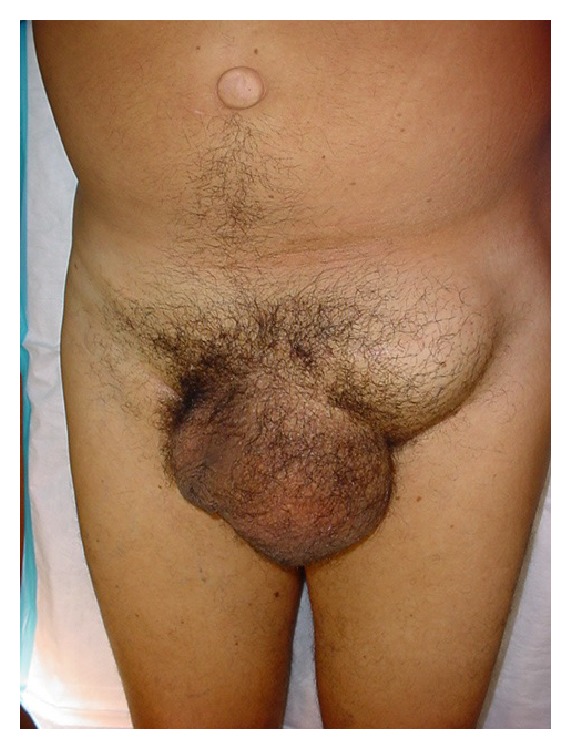
Clinical image of the left inguinoscrotal hernia mistakenly thought to be a bulge from contraband substances implanted subcutaneous by TSA security personnel after backscatter scanning.

A 68-year-old man had a left inguinoscrotal hernia that was asymptomatic and easily reducible (Figure 1). Despite the hernia, he was active and comfortably managed his retired lifestyle. At surgical consultation, he was advised with his options and chose not to have surgery. He was content managing the hernia conservatively for five years.

While traveling on holiday, he made an in-transit stop in a United States airport where he was required to enter a security scanner. Immediately upon exiting the scanner, he was approached by security personnel and rigorously questioned about the presence of a concealed item in his under garments. His explanation that he had an inguinal hernia was not accepted. In the presence of many onlookers at the busy airport, he was separated from his wife and escorted away in the custody of two armed airport security personnel.

After another elaborate round of questioning in an interrogation room, two additional officers were summoned, and the patient was subjected to a humiliating examination of the genitalia. Only after this prolonged exercise was he released back into the airport, resulting in a delay in his travels and ruining his vacation.

Frustrated, embarrassed, and inconvenienced, the patient returned home and immediately sought surgical consultation for inguinal herniorrhaphy. Although he managed his hernia conservatively for five years without event, he was now fearful of a repetition of this experience—this was his justification for surgical repair. Inguinal herniorrhaphy was completed uneventfully as an ambulatory case under general anaesthesia.

## 3. Discussion

Airport scanners were first introduced in Schipol Airport, Amsterdam in 2007. However, their widespread use in North America was delayed primarily due to the public's concerns about possible carcinogenic effects and invasion of privacy [3–5]. On Christmas day in 2009, Umar Abdulmutallab passed through airport security with explosives concealed in his under garments and boarded an airliner bound for Detroit [6, 7]. Although his terrorist plot to blow up the airliner was thwarted by passengers, he undoubtedly contributed to the stringent airport security measures existing today. A major resultant change was the introduction of scanners into United States airports in autumn 2010 [6]. After trials in Atlanta, Las Vegas, and Washington DC airports, their use as primary screening modalities became more widespread by July 2011 [7, 8].

These scanners use a technique called backscatter imaging where low intensity X-ray beams are used to create images [3–5]. Typically, the patient stands in a booth between two large receiver boxes. A generator within the booth directs low intensity X-ray beams toward the traveller. These are only strong enough to penetrate through clothing and a few millimetres into the body [4]. Reflected beams are captured by the large detectors flanking the traveller's bay and used to produce an image of the body.

Backscatter imaging technology was used in prisons for many years before being introduced in airports. Steven Smith, who developed and patented backscatter technology in 1991 [9], claims that it provides the same degree of detection capability as frisking [10]. It can detect weapons and/or contraband on the skin or in clothing, although it is less useful to detect items within body cavities [10]. This is because backscatter X-rays differ from transmission X-rays used in medical imaging which are strong enough to pass through the body to be received by a detector on the opposite side [7].

The United States Transportation Security Administration (TSA) reports that advanced imaging technology is successful in detecting nonmetal threats, including explosives and weapons [11]. Although the TSA reports that over 300 dangerous or illegal items were detected on passengers in US airports over one year [12], there are still groups opposed to the widespread use of airport scanners. Critics take issue with two points related to airport scanner use: the carcinogenic effect of exposure to ionizing radiation and invasion of privacy.

Although concerns about carcinogenesis have been raised, most existing data suggests that the small amount of radiation a traveller is exposed to from a backscatter scan is clinically insignificant [7, 13–17]. The radiation exposure from transmission X-rays during a single chest radiograph is approximately 1000 times greater than the exposure during a backscatter scan [4]. Direct measurements reveal that the dose of radiation from a single backscatter scan is extremely small, ranging from 0.015 *μ*Sv [13, 14] to 0.2 *μ*Sv [17]. This is similar to the dose of cosmic radiation absorbed during 2 minutes of flight [13] or 3–9 minutes of normal daily living [14]. The American Medical Association [17] released the statement “as of June 2013, no data exist to suggest that individuals, including those who are especially sensitive to ionizing radiation, should avoid backscatter security scanners due to associated health risks.”

The concerns over privacy stem from the detailed three-dimensional images of a traveller's body that are produced by the scanners [1, 5]. Privacy groups have likened these to nude images that can be kept in records along with identifying information for individual travellers. They also raise the point that there is inadequate control over who views the images and there is potential for unauthorized distribution. The TSA sought to appease privacy groups with two responses [18]: officers viewing the scanned imagery were moved to remote locations away from the passengers being scanned, and passengers were given the option to request an alternative form of screening as in the form of a “pat down.” The remote viewing did not solve the problem because detailed images were still being generated along with identifying information, and the fact that images were transmitted electronically compounded the potential for misuse. Additionally, there was also the concern that a “pat down” was not a good option as it could also be abused.

The debate heightened in July 2010 when the Electronic Privacy Information Centre (EPIC) petitioned the District of Columbia Circuit Court of Appeals to challenge the TSA's decision to use body scanners as the primary screening technique in US airports [19]. EPIC sought for the body scanner program to be suspended on the grounds that it violated several United States laws including the Administrative Procedures Act, the Privacy Act, the Religious Freedom Restoration Act, the Video Voyeurism Prevention Act, and the Fourth Amendment [19].

On July 15, 2011, the DC Circuit Court of Appeals ruled that the TSA violated the Administrative Procedures Act and ordered the agency to undertake the proper rule-making procedures and allow the public to comment on the body scanner program [19]. The US congress then ruled that beginning June 1, 2012, the TSA should only use advanced imaging technology (including backscatter scanners) equipped with automatic target resolution (ATR) software to address privacy concerns [20]. The ATR software produces a generic image of the individual being screened that is the same as the images produced for all other screened individuals [18–20]. These have been milestone rulings that will shape the use of advanced imaging technology and have initiated a move away from backscatter scanning toward millimeter radiowave machines with ATR software.

While the issues of privacy and exposure have been thoroughly debated in the literature, there is one concern that has not received due attention: the ill effect on common medical conditions. Although these scans are effective in detecting items on the skin or in clothing, they could miss items deep in a body cavity where the X-rays do not reach. Therefore, they could detect an abnormality that produces an aberration in the normal body contour such as an inguinal hernia, as happened in our patient. Because of the very limited tissue penetration, the examining officer would have difficulty in determining if the passenger had something inserted in a subcutaneous position. Our patient had an inguinoscrotal hernia but it could also happen with several other relatively common medical conditions such as sebaceous cysts, lipomata, fracture callus, medical implants, and dialysis grafts. Furthermore, if operators of advanced imaging technology in airports do not recognize these as medical conditions then travellers could be needlessly exposed to unnecessary scrutiny, embarrassment, and delayed movement as happened in our patient. In our patient, this was so significant that it prompted him to accept the risks [21] of an arguably unnecessary operation.

## 4. Conclusion

The use of advanced imaging technology at international airports is increasing in popularity as a corollary to heightened security concerns across the globe. Operators of airport scanners should be educated about common disorders, such as inguinal herniae, in order to avoid unnecessary harassment of travelers since they will encounter these with increasing frequency.
